# Productive Homologous and Non-homologous Recombination of Hepatitis C Virus in Cell Culture

**DOI:** 10.1371/journal.ppat.1003228

**Published:** 2013-03-28

**Authors:** Troels K. H. Scheel, Andrea Galli, Yi-Ping Li, Lotte S. Mikkelsen, Judith M. Gottwein, Jens Bukh

**Affiliations:** 1 Copenhagen Hepatitis C Program (CO-HEP), Department of Infectious Diseases and Clinical Research Centre, Copenhagen University Hospital, Hvidovre, and Department of International Health, Immunology and Microbiology, Faculty of Health and Medical Sciences, University of Copenhagen, Copenhagen, Denmark; 2 Laboratory of Virology and Infectious Diseases, Center for the Study of Hepatitis C, The Rockefeller University, New York, New York, United States of America; Yale University, United States of America

## Abstract

Genetic recombination is an important mechanism for increasing diversity of RNA viruses, and constitutes a viral escape mechanism to host immune responses and to treatment with antiviral compounds. Although rare, epidemiologically important hepatitis C virus (HCV) recombinants have been reported. In addition, recombination is an important regulatory mechanism of cytopathogenicity for the related pestiviruses. Here we describe recombination of HCV RNA in cell culture leading to production of infectious virus. Initially, hepatoma cells were co-transfected with a replicating JFH1ΔE1E2 genome (genotype 2a) lacking functional envelope genes and strain J6 (2a), which has functional envelope genes but does not replicate in culture. After an initial decrease in the number of HCV positive cells, infection spread after 13–36 days. Sequencing of recovered viruses revealed non-homologous recombinants with J6 sequence from the 5′ end to the NS2–NS3 region followed by JFH1 sequence from Core to the 3′ end. These recombinants carried duplicated sequence of up to 2400 nucleotides. HCV replication was not required for recombination, as recombinants were observed in most experiments even when two replication incompetent genomes were co-transfected. Reverse genetic studies verified the viability of representative recombinants. After serial passage, subsequent recombination events reducing or eliminating the duplicated region were observed for some but not all recombinants. Furthermore, we found that inter-genotypic recombination could occur, but at a lower frequency than intra-genotypic recombination. Productive recombination of attenuated HCV genomes depended on expression of all HCV proteins and tolerated duplicated sequence. In general, no strong site specificity was observed. Non-homologous recombination was observed in most cases, while few homologous events were identified. A better understanding of HCV recombination could help identification of natural recombinants and thereby lead to improved therapy. Our findings suggest mechanisms for occurrence of recombinants observed in patients.

## Author Summary

Genetic recombination is the alternative joining of nucleic acids leading to novel combinations of genetic information. While DNA recombination in cells is of importance for evolution and adaptive immunity, RNA recombination often has only transient effects. However, RNA viruses are rapidly evolving and recombination can be an important evolutionary step in addition to mutations introduced by the viral polymerase. Recombination can allow escape from the host immune system and from antiviral treatment, and recombination of live attenuated viral vaccines has led to re-emergence of disease. Hepatitis C virus (HCV) is an important human pathogen that chronically infects more than 130 million worldwide and leads to serious liver disease. For HCV, naturally occurring recombinants are rare but clinically important. HCV recombination constitutes a challenge to antiviral treatment and can potentially provide an escape mechanism for the virus. In this study, we established an assay for HCV RNA recombination and characterized the emerging homologous and non-homologous recombinant viruses. Interestingly, recombination did not depend on viral replication, occurred most efficiently between isolates of the same genotype and did not occur with strong site-specificity. Better diagnosis of clinically important recombinants and an increased knowledge on viral recombination could strengthen antiviral and vaccine development.

## Introduction

RNA viruses are rapidly adapting to their environment. The error-prone viral polymerases and the lack of proofreading mechanisms for most RNA viruses lead to high mutation rates. Genetic recombination between viral genomes is an additional mechanism increasing genetic diversity, which has proven to be epidemiologically relevant and allows RNA viruses to adapt to their surroundings [1]. Recombination could allow escape from natural or therapeutically induced immunity [2], or during antiviral treatment constitute an escape mechanism to antiviral compounds with an otherwise high barrier to resistance [3]. In addition, viral recombination has been associated with increased pathogenicity [4], and has caused the emergence of new human pathogens, such as Western equine encephalitis virus [5]. The use of live attenuated viral vaccines has led to re-emergence of disease due to recombination of vaccine strains with related viruses [6], [7]; this remains a problem in poliovirus eradication. Thus, understanding the nature of viral recombination has general evolutionary implications, and might affect treatment and vaccination for important human pathogens.

Significant differences have been reported in recombination frequencies for different virus families, with high frequencies among *Picornaviridae* and lower frequencies among *Flaviviridae* and *Alphaviridae* [8]. Although hepatitis C virus (HCV) belongs to the *Flaviviridae* family, several epidemiologically important recombinant strains have been reported [9]–[11]. HCV constitutes a major public health burden with 130–170 million people chronically infected. Infection leads to increased risk of hepatitis, liver cirrhosis and hepatocellular carcinoma. The single positive-stranded HCV RNA genome of around 9600 nucleotides encodes one long open reading frame (ORF) flanked by 5′ and 3′ untranslated regions (UTRs). The HCV polyprotein is co- and post-translationally processed into structural (Core, E1 and E2), and nonstructural proteins (p7, NS2, NS3, NS4A, NS4B, NS5A and NS5B). Significant diversity is found among HCV isolates, which are grouped into seven major genotypes and many subtypes [12]. Genotypes, subtypes and isolates/strains differ at around 30%, 20% and 2–10%, respectively, at the nucleotide and amino acid levels.

The epidemiologically most important HCV recombinant is the homologous recombinant of genotype 2k/1b that was first identified in St. Petersburg [13]. Since then, a number of naturally occurring inter- and intra-genotypic recombinants have been reported [9]–[11]; most inter-genotypic recombinants have junction in or close to the NS2 gene. Further, naturally occurring subgenomic deletion mutants have been described to persist in around 20% of patients [14], [15]. The prevalence of recombinants might be underestimated due to lack of routine screening; in addition, recombination events between isolates of the same subtype could be difficult to identify and distinguish from new isolates [16]. While mechanisms and kinetics remain problematic to study in patients, in vitro systems could provide a better understanding of HCV recombination, leading to improvements in detection of natural recombinants.

Treatment with interferon-α and ribavirin leads to sustained viral response for only around half of HCV infected patients, and many cannot be treated due to side effects or contraindications. The recent approval of novel directly acting antiviral compounds is expected to increase successful treatment rates. Great HCV genotype-specific differences exist in the outcome of antiviral therapy, and in the recommended treatment regimens [17], [18]. Thus, genotyping from a single gene region could mislead decisions on treatment regimens for recombinant viral strains. In addition, RNA recombination could function as an escape mechanism to therapy with novel directly acting antiviral compounds.

Two possible mechanisms of RNA recombination are generally considered for RNA viruses: replicative and non-replicative. In the replicative copy-choice mechanism, the viral polymerase changes template during RNA synthesis whereas in the non-replicative mechanism, RNA breakage and rejoining occur. Both mechanisms can in principle lead to homologous and non-homologous recombinants. The copy-choice mechanism is the best characterized [1], [19], [20], and was first described for poliovirus [21]. Productive non-replicative recombination was so far only demonstrated in few studies on poliovirus [22] and bovine viral diarrhea virus (BVDV) [23], which belongs to the HCV-related pestiviruses.

Few experimental studies have investigated recombination of HCV, and our understanding of its mechanisms is still limited. One study examined HCV recombination in co-infected chimpanzees and identified homologous recombinants between genotypes 1a and 1b [24]. In another recent study, recombination frequency was investigated using the bicistronic selectable HCV replicon system [25]. In the present study, we aimed at investigating the nature of HCV recombination in infectious cell culture systems.

## Results

### Co-transfection of HCV genomes lacking viability *in vitro* led to productive non-homologous recombination

**Figure 1:**
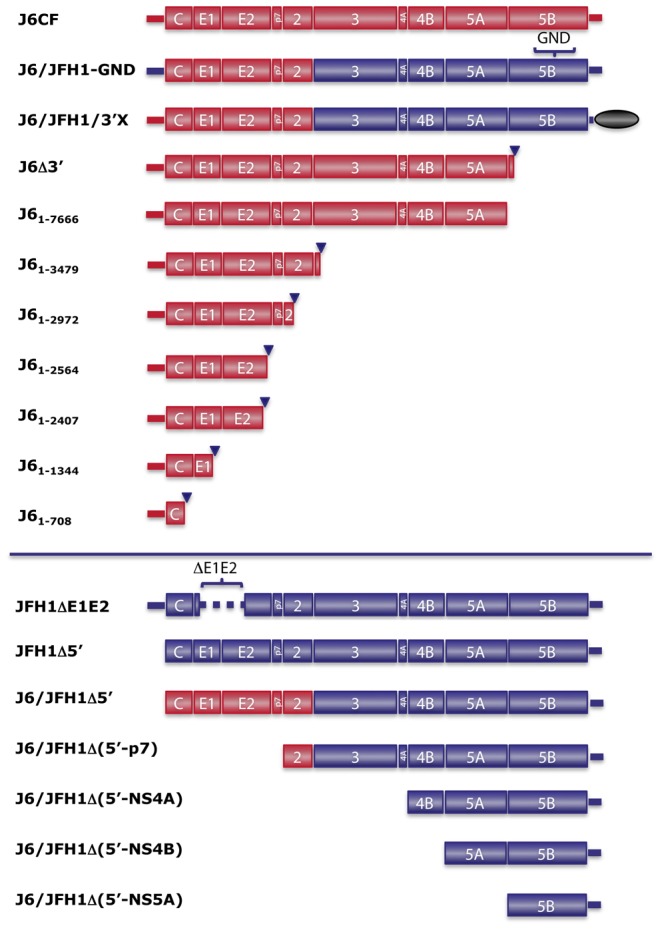
HCV genomes of strains J6 and JFH1 used for co-transfection experiments in the recombination assay. Genomes from the top panel were co-transfected with genomes from the bottom panel. Genomes are color coded according to isolate (J6: red, JFH1: blue). The black oval indicates replacement of 3′UTR sequence by an irrelevant cellular RNA sequence. Triangle denotes cleavage of pJ6CF by restriction enzyme; where no triangle is indicated plasmids were constructed with the HCV sequence shown. Details of individual genomes are given in Materials & Methods.

To study HCV recombination, an assay was established using the Huh7.5 hepatoma cell line. Since recombination of HCV is thought to be a relatively rare event, HCV genomes lacking viability *in vitro* were co-transfected to facilitate the identification of viable recombinants. RNA transcripts of the JFH1ΔE1E2 genome were transfected alone or in combination with either the J6CF or J6/JFH1-GND genome (all genotype 2a, Figure 1). JFH1ΔE1E2 carries a partial deletion of the envelope genes, which allows replication but not viral particle production. The consensus full-length clone of the J6 isolate, J6CF, does not replicate in Huh7.5 cells [26] but has a functional 5′UTR-NS2 region *in vitro* [27], while the replication-deficient J6/JFH1-GND, carries an NS5B polymerase mutation in the viable J6/JFH1 background [28].

**Figure 2:**
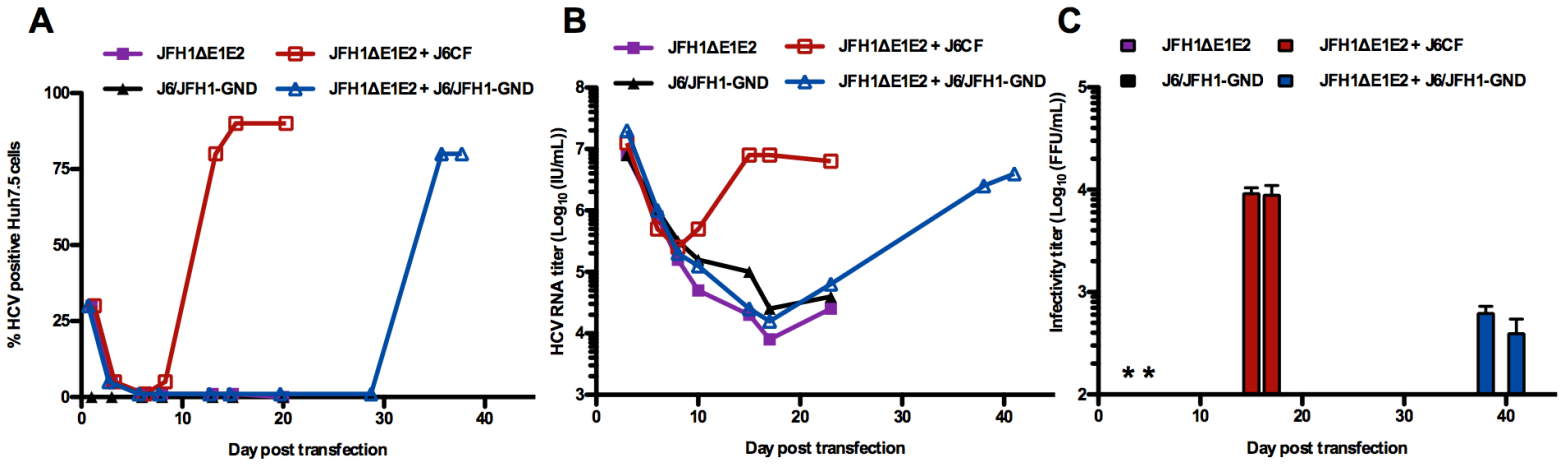
Co-transfection of JFH1ΔE1E2 and replication deficient genomes into Huh7.5 cells. HCV genomic RNA transcripts of JFH1ΔE1E2 were transfected alone or in combination with J6CF or J6/JFH1-GND. In addition, J6/JFH1-GND was transfected alone as a replication negative control. Cultures were followed until day 23, at which time the JFH1ΔE1E2 control had become negative; co-transfection of JFH1ΔE1E2 and J6/JFH1-GND was followed until day 41 and never became negative. (**A**) Percentage of HCV Core positive cells as determined by immunostainings. No positive cells were observed when J6/JFH1-GND was transfected alone. (**B**) HCV RNA titers (IU/mL) in supernatant after transfection. (**C**) Infectivity titers (FFU/mL) in supernatant after transfection. *Titrations were negative for all cultures on day 3 and 6. Other time points were not measured.

In all experiments, around 30% of cells were positive for HCV Core one day after transfection (Figure 2A); this percentage rapidly decreased due to lack of spread of infection and growth advantages of untransfected cells, as previously shown [29]. HCV RNA levels in the supernatant were comparable for all cultures during the first 8 days (Figure 2B) and no infectious particles were released from any of the cultures on day 3 and 6 (Figure 2C). An increase in percentage of HCV positive cells and HCV RNA levels was observed for the culture co-transfected with JFH1ΔE1E2 and J6CF from day 10 and infection spread to the almost entire culture on day 13. Similarly, infection spread to the majority of cells around day 36 in the culture co-transfected with JFH1ΔE1E2 and J6/JFH1-GND. After spread of infection in culture, infectivity titers of around 10^4^ focus-forming units (FFU)/mL or 10^3^ FFU/mL, respectively, were observed in supernatant from the two cultures (Figure 2C). After passage of supernatant from the J6CF co-transfected culture to naïve cells, HCV RNA titers above 10^7^ IU/mL and infectivity titers around 10^4^ FFU/mL were produced. Two additional co-transfections of JFH1ΔE1E2 and J6CF led to similar results, with spread of infection to the majority of the culture after 8 and 25 days, respectively.

**Figure 3:**
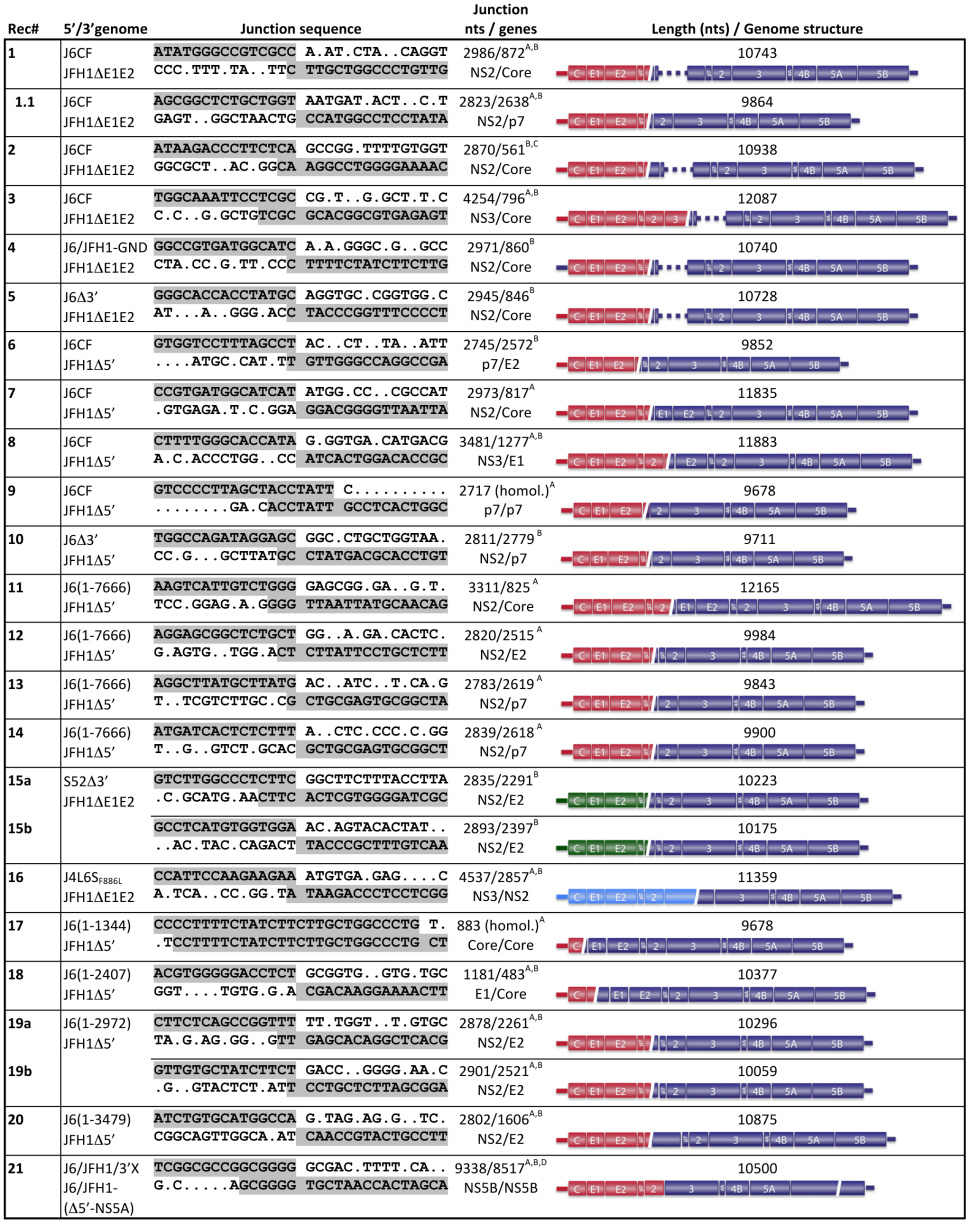
Characteristics of recombined HCV genomes. For each observed recombination event (Rec#), the 30 nt sequence around the recombination breakpoint is shown for the parental 5′ and 3′ genomes. Grey shading indicates the sequence of the recombined genome. Conserved nucleotides around the junction site are shown as dots. In cases where breakpoints were located at stretches of conserved nucleotides in the two parental sequences, numbering is consistently done to include most of the 5′ fragment and is indicated by space separation of the sequence. Homologous (homol.) recombination events are indicated. The predicted total genome length is given, assuming that the recombination breakpoint was the only recombination event present. Schematic drawings of the genome structure of individual recombinants are shown. ^A^ Junction identified by direct sequencing of PCR products. ^B^ Junction identified by sequencing of cloned fragments. ^C^ One of seven clones contained an in-frame deletion of JFH1ΔE1E2 nt 926–957. ^D^ The same junction was subsequently also found for co-transfections of J6/JFH1Δ5′, J6/JFH1Δ(5′-p7) and J6/JFH1Δ(5′-NS4A).

To determine the nature of the infectious HCV genomes from the original co-transfection of JFH1ΔE1E2 with J6CF after passage to naïve cells, we performed direct sequencing of 12 overlapping PCR amplicons covering the entire ORF. While amplicons 1–2 (5′UTR-E2) had J6 sequence, amplicons 3–12 (E2-3′UTR) had JFH1 sequence; amplicons 2 and 3 contained overlapping sequence in E2 from both strains, which indicated the presence of a duplicated region. This was further analyzed for all three cultures co-transfected with JFH1ΔE1E2 and J6CF by cloning of longer PCR amplicons and amplicons generated by inverted primer sets. The resulting sequences revealed non-homologous recombinant genomes with different genomic structures. The first recombinant had J6 sequence from the 5′UTR to nucleotide (nt) 2986 (NS2), recombined with JFH1ΔE1E2 from nt 872 (Core) to the 3′UTR (Rec#1; including the envelope deletion from nt 991 to 2040) (Figure 3). This recombination produced an in-frame non-homologous recombinant HCV ORF containing 1065 duplicated nts (355 amino acids) with a total predicted genome length of 10743 nts, compared to 9678 for JFH1 and 9711 for J6CF. A second recombinant had J6 sequence from the 5′UTR to nt 2870 (NS2), recombined with JFH1ΔE1E2 at nt 561 (Core) (Rec#2) (Figure 3). The third recombinant had breakpoint further downstream with J6 sequence from the 5′UTR to nt 4254 (NS3) joined to JFH1ΔE1E2 from nt 796 (Core) (Rec#3). The resulting genome had a predicted length of more than 12 kb, over 2400 nucleotides longer than natural HCV isolates. While this is longer than typical infectious HCV reporter constructs expressing fluorescent or luminescent markers [30], much longer BVDV recombinants (up to around 20 kb) were identified in similar cell culture recombination experiments [23].

It was previously demonstrated that the NS3 helicase contributes to the unique replication abilities of the JFH1 isolate [31]. Since this might have restricted the region of recombination in co-transfections of JFH1ΔE1E2 and J6CF, we investigated whether a different type of recombination event had occurred in the culture co-transfected with JFH1ΔE1E2 and J6/JFH1-GND, where both genomes carried an NS3 protein of JFH1 origin. After passage of viral supernatant to naïve cells, sequencing of the entire ORF from recovered viruses again showed J6 sequence for amplicons 1–2 and JFH1 sequence for amplicons 3–12. In further analysis, PCR amplicon clones covering the junction revealed a recombinant genome with J6/JFH1-GND sequence from the 5′UTR to nt 2971 (NS2), followed by JFH1ΔE1E2 from nt 860 (Core) to 3′UTR (Rec#4) (Figure 3), similar in structure to those already identified.

### Recombination does not depend on a functional HCV polymerase

In the initial recombination assay, a replicating genome (JFH1ΔE1E2) was co-transfected with a non-replicating genome (J6CF or J6/JFH1-GND). To determine whether putative low-level replication of J6CF or replication of J6CF in trans by the JFH1 replicase played a role in recombination, we co-transfected JFH1ΔE1E2 with J6Δ3′. J6Δ3′ was produced by linearization of the DNA in the beginning of NS5B and would therefore not express the polymerase or carry a 3′UTR (Figure 1). This experiment led to results similar to co-transfections of JFH1ΔE1E2 with J6CF, with spread of infection to the majority of the culture after 13 days. After passage to naïve cells, sequencing of the replicating genome demonstrated a junction from NS2 of J6Δ3′ to Core of JFH1ΔE1E2 (Rec#5, Figure 3). Thus, a functional J6 polymerase and a complete 3′UTR was not a requirement for recombination, which apparently did not depend on replication of both genomes.

To determine whether at least one functional HCV polymerase would be required for recombination, we co-transfected two non-replicating genomes. Four replicate co-transfections were performed using J6CF, which is unable to replicate *in vitro*, and JFH1Δ5′, which lacks the entire 5′UTR and therefore cannot undergo translation or replication (Figure 1), such that no viral replication could occur in the transfected cells. In addition, JFH1Δ5′ was co-transfected with J6Δ3′ (one replicate) or with transcripts from the pJ6_1–7666_ plasmid (four replicates), which was constructed to only contain J6 5′UTR-NS5A sequence, thus ensuring that no polymerase protein was produced (Figure 1). In these experiments, no or very few HCV positive cells were observed by immunostaining one day after transfection. However, infection emerged in few cells in all cultures by day 4 and spread to the majority of all nine cultures in 10–32 days. After passage to naïve cells, replicating genomes were characterized by sequencing. Three of the four recombinants from the cultures co-transfected with complete J6CF genomes had structures similar to those identified in the JFH1ΔE1E2 co-transfections; one had junction from p7 to E2 (Rec#6), another from NS2 to Core (Rec#7), and the third from NS3 to E1 (Rec#8). Interestingly, the last recombination event was homologous with breakpoint between nt 2710–2717 in p7 (Rec#9) (Figure 3). In the culture co-transfected with J6Δ3′, we identified a heterologous recombinant with a short duplication of just 33 nts and junction from nt 2811 (NS2) to nt 2779 (p7) (Rec#10) (Figure 3). Heterologous recombinants were also observed in all four cultures after co-transfection with J6_1–7666_, with junctions from NS2 to Core (Rec#11), from NS2 to E2 (Rec#12) or from NS2 to p7 (Rec#13 and Rec#14) (Figure 3).

**Figure 4:**
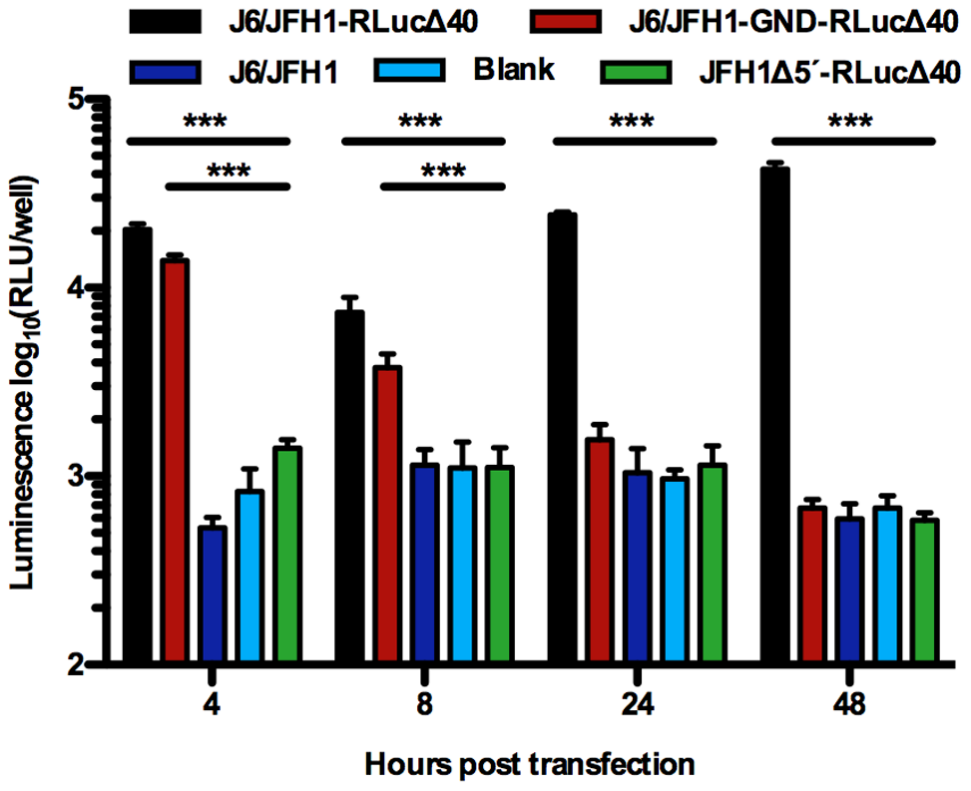
Measurement of translation from input RNA. To evaluate translation from input JFH1Δ5′ RNA using luciferase reporter genomes, Huh7.5 cells were transfected with JFH1Δ5′-RLucΔ40, J6/JFH1-RLucΔ40 (positive control for translation and replication), J6/JFH1-GND-RLucΔ40 (positive control for translation, negative control for replication) and J6/JFH1 (replicating, negative control for luciferase expression). Relative light units (RLU) of Renilla luminescence were measured at indicated time points and the mean and standard error of the mean of five replicates are shown. Differences in signal intensities at the individual time points were evaluated statistically using ANOVA with Bonferroni correction. Highly significant (p<0.0001) differences to JFH1Δ5′-RLucΔ40 levels are indicated (***), other differences to JFH1Δ5′-RLucΔ40 were not significant.

To validate that no translation was occurring from JFH1Δ5′ leading to the presence of HCV polymerase, we generated a JFH1Δ5′-RLucΔ40 reporter construct with renilla luciferase inserted into NS5A [30], and measured low-level translation from transfected input RNA in luciferase assays. In measurements from 4–48 hours post transfection luciferase signals were observed for the positive control, J6/JFH1-RLucΔ40, and 4–8 hours after transfection for J6/JFH1-GND-RLucΔ40, for which translation but not replication could occur. In contrast, signals for JFH1Δ5′-RLucΔ40 were comparable to the background signal for all time points (Figure 4). Thus we concluded that a functional HCV polymerase was not required for recombination to occur in cell culture.

### Viability of non-homologous recombinants confirmed by reverse genetic studies

**Figure 5:**
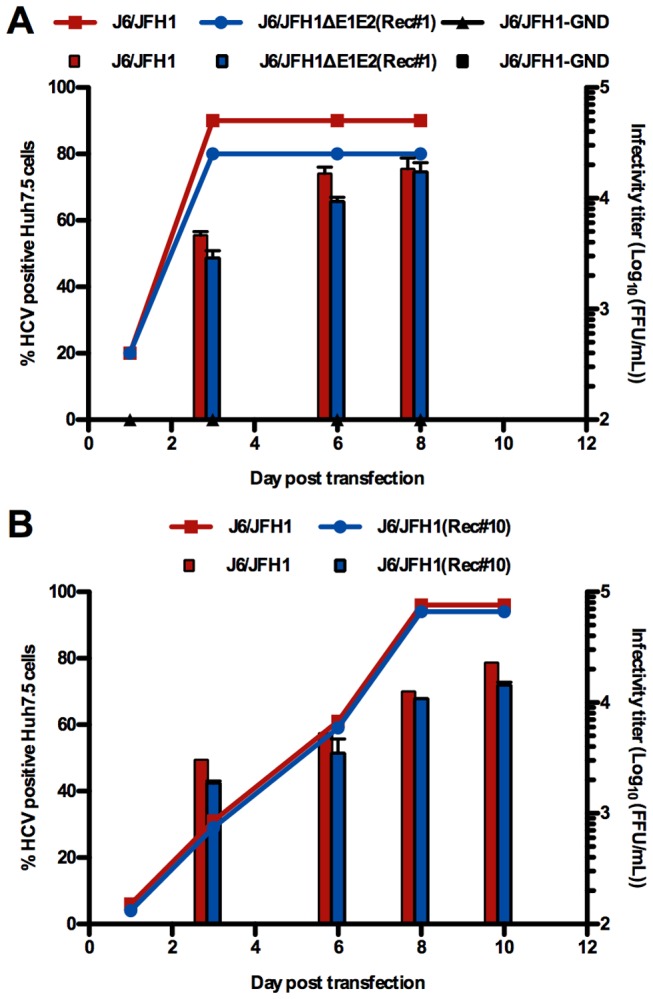
Transfection of the cloned recombinants J6/JFH1ΔE1E2(Rec#1) (A), or J6/JFH1(Rec#10) (B) in Huh7.5 cells. HCV genomic RNA transcripts were transfected and compared to J6/JFH1. The J6/JFH1-GND control remained negative throughout the experiment shown in (A). Percentage of HCV Core positive cells as determined by immunostainings (lines) and viral infectivity titers measured in supernatant (bars) are shown.

To confirm that the identified non-homologous recombinants were viable, two representative clones, J6/JFH1ΔE1E2(Rec#1) and J6/JFH1(Rec#10) were generated based on the original J6CF, JFH1ΔE1E2 and JFH1 consensus clones. After transfection into Huh7.5 cells, J6/JFH1ΔE1E2(Rec#1) and J6/JFH1(Rec#10) immediately spread in culture and produced infectivity titers greater than 10^4^ FFU/mL (Figure 5). Similar infectivity titers were produced after passage of J6/JFH1ΔE1E2(Rec#1) and J6/JFH1(Rec#10) supernatant to naïve cells. Sequencing of the entire ORF confirmed the identity of the replicating recombinants. J6/JFH1(Rec#10) did not acquire mutations, while J6/JFH1ΔE1E2(Rec#1) had acquired A2071S and C2574R (A1712S and C2215R according to the H77 reference polyprotein, AF009606). These changes were not observed from the original co-transfected culture. Thus, the recombined genomes were fully viable in cell culture and the initially identified genomic structures were confirmed.

### Sequential recombination events observed after serial passage in culture

**Figure 6:**
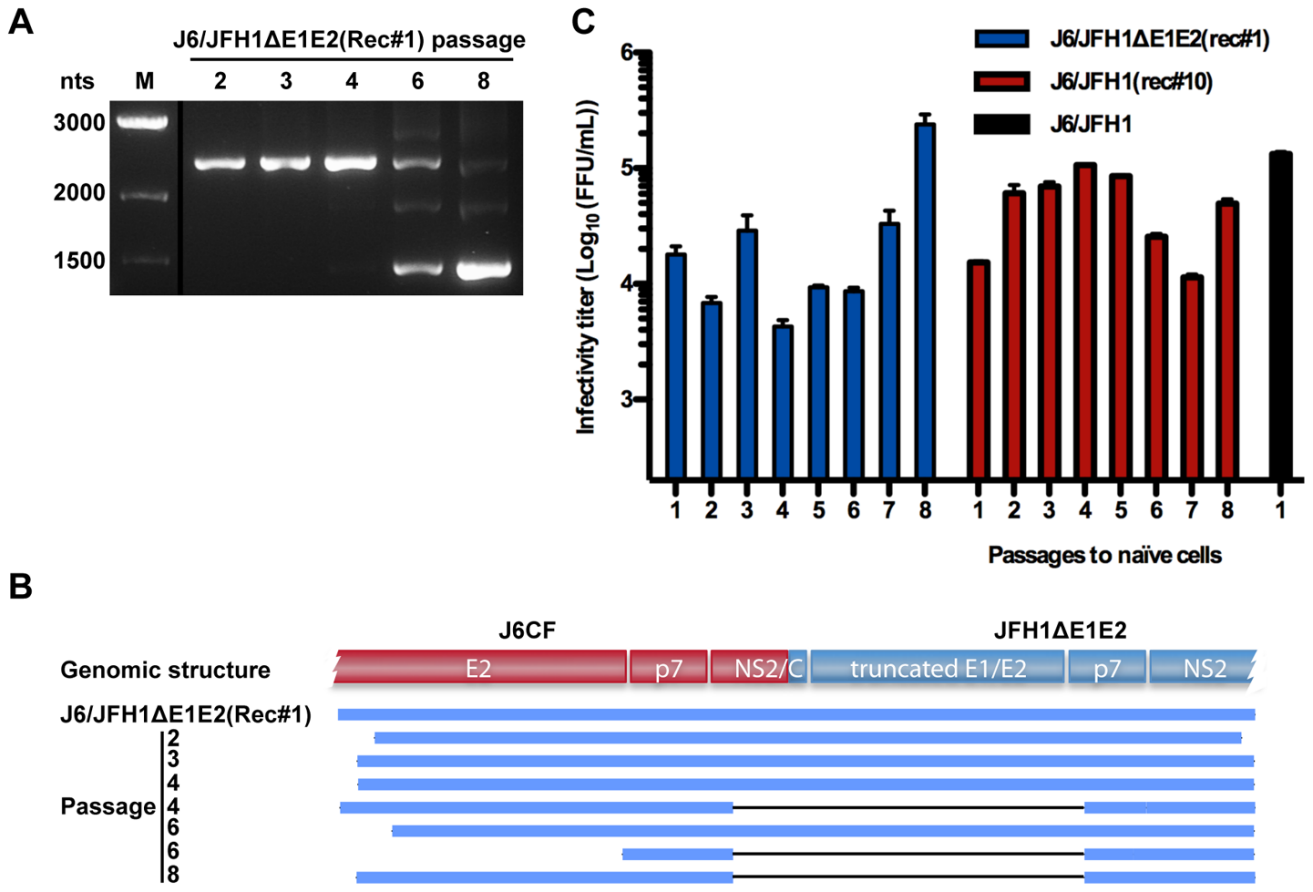
Characterization of sequential recombination events. After long-term passage in Huh7.5 cell culture a second sequential recombination event occurred for J6/JFH1ΔE1E2(Rec#1) but not for J6/JFH1(Rec#10). (**A**) PCR validation of the recombination region of Rec#1. A PCR was designed to cover the primary and secondary recombination events (see Materials & Methods). A Rec#1 type junction yielded an amplicon of 2321 nts (evident until passage 6), while a Rec#1.1 type junction yielded an amplicon of 1442 nts (evident from passage 6 onwards, and as early as passage 3 on long exposure images). Exact recombination sites are given in Figure 3. M, size marker. No size change was observed for amplicons covering the Rec#10 junction. (**B**) Schematic overview of recombinant types found in the original co-transfection experiment (J6/JFH1ΔE1E2(Rec#1)) and in passage 2–8 of the cloned Rec#1 to naïve cells. Regions within the PCR amplicon shown in (A) that were sequenced to reveal the recombinant junction are shown with blue bars; gaps (deletions) are shown with black lines. The genome structure included NS2/Core and E1/E2 fusion proteins for the original Rec#1 and an NS2/p7 fusion protein after the second recombination event. (**C**) Peak infectivity titers in serial passage of J6/JFH1ΔE1E2(Rec#1) and J6/JFH1(Rec#10) in culture. A representative titer after infection of naïve cells (passage 1) with J6/JFH1 is shown for comparison.

To determine whether sequential recombination events could occur on the same genome, we performed long term passaging of the J6/JFH1ΔE1E2(Rec#1) and J6/JFH1(Rec#10) recombinants by serial inoculation of naïve cells with supernatant from fully infected cultures. Interestingly, after three passages to naïve cells a novel recombinant was detected in the J6/JFH1ΔE1E2(Rec#1) culture. The genetic structure of the new genome showed that an additional non-homologous recombination event had taken place and removed most of the duplicated region, resulting in a junction from nt 2823 (NS2) of J6 to nt 2638 (p7) of JFH1 (Rec#1.1, Figure 3). This second-generation recombinant was detectable from passage 3 and dominated the virus population from passage 6 (Figure 6A and B). The peak supernatant infectivity titer increased in passage eight, where the shorter Rec#1.1 genome dominated (Figure 6A and C). In contrast, no changes occurred to the comparably short duplicated junction region of J6/JFH1(Rec#10) during 8 serial passages. Infectivity titers of almost 10^5^ FFU/mL were observed in most passages for this apparently genomically stable recombinant (Figure 6C). Thus, sequential recombination events could take place in culture to eliminate long duplicated and presumably non-functional genome regions, apparently leading to increase of viral fitness.

### Evaluation of recombination frequency between isolates of the same genotype

**Figure 7:**
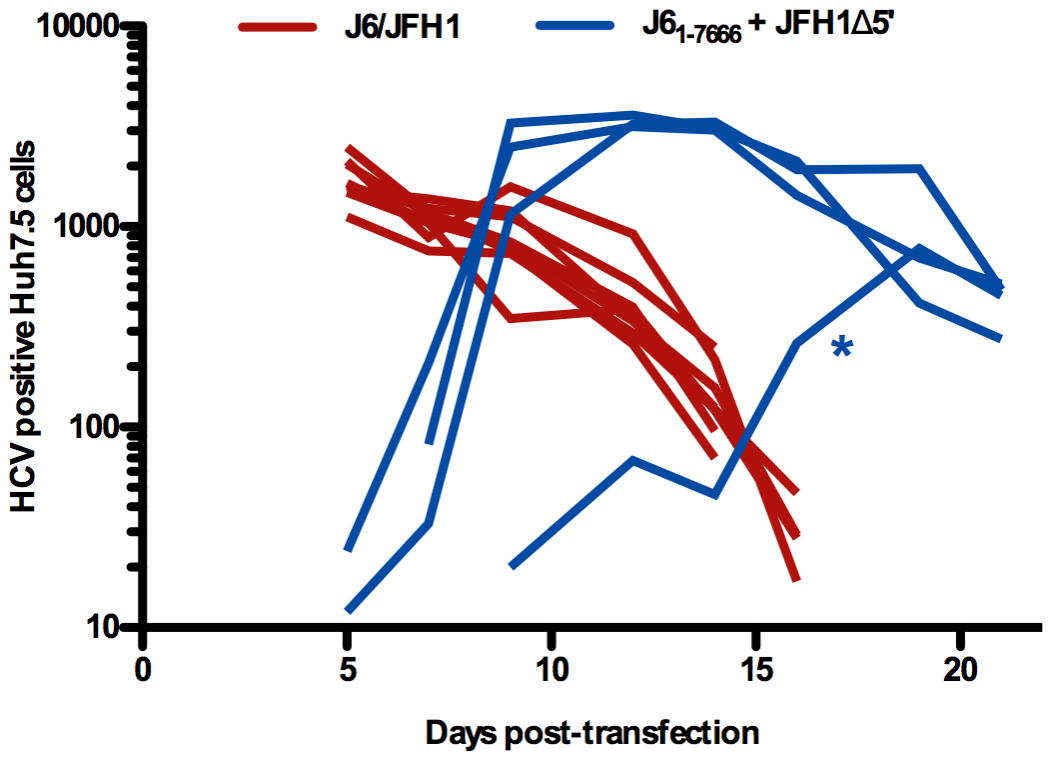
Emergence of positive recombinants in frequency experiment. Cells were transfected and 18 hours later distributed into 96-well format (7000 cells plated per well) to study recombination frequency. The number of HCV positive cells per well of replica staining plates plated ever 2–3 days (as indicated in Materials & Methods) was followed over time and is shown for the 8 J6/JFH1 positive controls and the 4/72 wells co-transfected with J6_1–7666_ and JFH1Δ5′, where recombinants emerged. Contamination of these four cultures by J6/JFH1 was excluded by passaging of virus to naïve cells and sequencing the NS2/NS3 junction, except for one recombinant (*) that was too attenuated to efficiently re-infect naïve cells. Cell numbers below 10, corresponding to background, are not plotted. Decline in number of infected cells correlated with massive virus induced cell death.

All 14 co-transfection experiments with J6 and JFH1-based genomes performed so far led to emergence of viable recombinants. To get a more quantitative understanding of recombination frequencies we re-plated cells co-transfected with JFH1Δ5′ and J6_1–7666_ into 96-well format before virus production was expected to occur. This would allow an estimation of recombination frequency between the genotype 2a isolates J6 and JFH1 over the Core-NS5A region. Through 22 days of follow up, 8 controls transfected with J6/JFH1 were positive, while recombination occurred in 4/72 (5.6%) co-transfected wells (Figure 7). Taking into account that 7000 cells were plated per well and that the transfection efficiency was 50% (assuming that co-transfection had the same efficiency as observed when evaluating NS5A positive cells one day post transfection of J6/JFH1) this equals to one productive recombination event for every 63,000 co-transfected cells, or recombination in 0.0016% of the cells.

### Infrequent recombination between isolates of different genotypes

**Figure 8:**
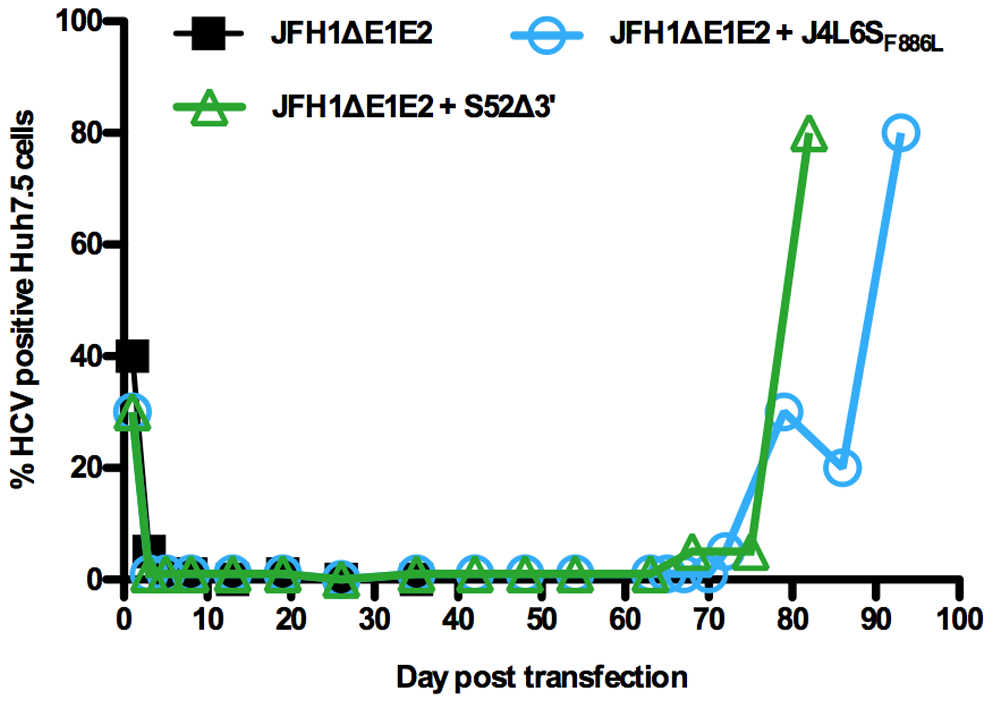
Co-transfection of JFH1ΔE1E2 and replication deficient genomes of other HCV genotypes into Huh7.5 cells. HCV genomic RNA transcripts of JFH1ΔE1E2 were transfected alone or in combination with S52Δ3′ or J4L6_SF886L_. Percentage of HCV Core positive cells as determined by immunostaining is shown. The JFH1ΔE1E2 culture was followed until day 35; no positive cells were observed after day 19 in this culture. For 16 other intergenotypic co-transfections, no infectious virus emerged and data similar to JFH1ΔE1E2 transfection alone were observed.

Intergenotypic recombinants were previously identified *in vivo* [9]–[11], and synthetic intergenotypic recombinants could establish infection in cell culture [32], [33]. Thus, we next investigated whether recombination *in vitro* could also occur between isolates of different genotypes. Since efficient replication in the infectious cell culture system at the outset of this study relied on the JFH1 isolate, we co-transfected JFH1ΔE1E2 with consensus clones of genotype 1a (H77C and HC-TN), 1b (J4L6S), 3a (S52) or 4a (ED43) or with 3′ truncated versions (truncation in NS5B) of the same genomes. Similarly to J6CF, these clones are infectious in chimpanzees but cannot replicate in Huh7.5 cells [26]. RNA transcripts of JFH1ΔE1E2 were co-transfected with H77C, HC-TN (3 replicates each), H77CΔ3′, HC-TNΔ3′, J4L6S, J4L6SΔ3′, S52, S52Δ3′, ED43 or ED43Δ3′ (1 replicate each). The percentage of HCV positive cells in most cultures was similar to transfection of JFH1ΔE1E2 alone, with a rapid decrease leading to no positive cells from around day 20. However, few HCV positive cells remained in the culture co-transfected with S52Δ3′ and infection eventually spread to the almost entire culture after 82 days (Figure 8). After passage of supernatant to naïve cells, cloning of PCR amplicons identified intergenotypic non-homologous recombination events. Of 13 clones, 6 contained S52 sequence until nt 2835 (NS2) and JFH1 sequence from nt 2291 (E2) (Rec#15a), while 7 clones had a slightly different junction between nt 2893 (NS2) of S52 and nt 2397 (E2) of JFH1 (Rec#15b) (Figure 3). While only two mutations were identified after passage in culture of the genotype 2a/2a recombinant Rec#1, direct sequencing of the almost entire ORF of the S52/JFH1 (3a/2a) recombinant identified a number of mutations, including coding mutations in Core, E1, E2, p7, NS4B and NS5A. This indicated a need for adaptive mutations for functional interaction of isolates from different genotypes.

We previously demonstrated that most synthetic JFH1 recombinants with genotype-specific Core-NS2 relied on adaptive mutations for efficient production of intracellular infectious particles [32], [34]. Since many recombination events identified in this study occurred in the NS2 region, we speculated that recombination between genomes carrying previously identified adaptive mutations might enhance the production of functional intergenotypic recombinants in our assay. We thus co-transfected JFH1ΔE1E2 with J4L6S_F886L_ or ED43_T827A,T977S_ that carried mutations previously shown to confer adaptation to the Core-NS2 recombinants, J4/JFH1 and ED43/JFH1 [32], [35]. While no recombination occurred in triplicate co-transfections with ED43_T827A,T977S_, co-transfection with J4L6S_F886L_ resulted in spread of infection to the majority of cells 93 days post-transfection (Figure 8). After passage to naïve cells, sequencing identified intergenotypic non-homologous recombination. The replicating genome contained J4L6S_F886L_ sequence from the 5′UTR to NS3 and JFH1 from NS2 to the 3′UTR, and carried the introduced mutation F886L (Rec#16, Figure 3). Thus, introduction of mutations conferring adaptation to synthetic intergenotypic JFH1-based Core-NS2 recombinants had only limited effect, on recombination frequency.

While all intragenotypic co-transfections performed with high input RNA led to emergence of viable recombinants, only two intergenotypic recombination events were identified from a total of 18 co-transfection experiments. Considering all co-transfection experiments with JFH1ΔE1E2 and different clones of other genotypes, an estimated generalized recombination frequency would be one productive recombination event per million co-transfected cells, or recombination in 0.0001% of the cells, taking into account two productive recombination events, the starting number of 400,000 cells in each of 18 experiments and an estimated average transfection efficiency of 30%.

### Recombination sites were not restricted to specific regions of the HCV genome

The recombination events identified so far all had breakpoints in the p7-NS3 region of the 5′ fragment and the Core-NS2 region of the 3′ fragment. Due to the lack of functional envelope genes in the JFH1ΔE1E2 construct, many recombination breakpoints were however restricted from occurring further upstream. Likewise, due to the importance of the NS3 helicase for the unique replication abilities of the JFH1 isolate [31], breakpoints could be restricted from occurring further downstream of non-JFH1 genomes. To investigate whether recombination events could occur in other regions, we co-transfected JFH1Δ5′ with versions of J6CF truncated at nt 708, 1344, 2407, 2564, 2972 or 3479 (Figure 1). While no spread of infection was identified in two cultures (J6_1–708_ and J6_1–2564_), the majority of cells in the other cultures became infected after 13–22 days. Recombined genomes were identified after passage to naïve cells. Another case of homologous recombination was identified in the culture co-transfected with J6_1–1344_, occurring in the nt 858–883 region (Core) (Rec#17). The three other recombination events were non-homologous with junctions from E1 to Core (Rec#18; J6_1–2407_), a mixed population of 2878 (NS2)/2261 (E2) and 2901 (NS2)/2521 (E2) (Rec#19a/b; J6_1–2972_), and from NS2 to E2 (Rec#20; J6_1–3479_) (Figure 3). Thus, recombination of J6 and JFH1 occurred outside the NS2 region, even in the most upstream gene, Core.

Next, we wanted to determine whether recombination could occur downstream of NS3. Since JFH1 exhibits efficient function of the NS3-NS5B region in Huh7.5 cells, we transfected 5′ truncated transcripts of J6/JFH1 together with J6/JFH1/3′X, which carried the 5′UTR-NS2 from J6CF, NS3-3′UTR(polyU) from JFH1 and an irrelevant human mRNA sequence replacing the 3′X region (Figure 1). No HCV positive cells were observed when any of these genomes were transfected alone. Thus, J6/JFH1/3′X was co-transfected with J6/JFH1Δ5′ lacking the 5′UTR, J6/JFH1Δ(5′-p7), J6/JFH1Δ(5′-NS4A), J6/JFH1Δ(5′-NS4B), or J6/JFH1Δ(5′-NS5A). While no productive recombination occurred in the J6/JFH1(Δ5′-NS4B) co-transfected culture, infection spread in all other cultures after 8–17 days. Interestingly, identical recombinants were identified after passage of virus from all four positive cultures to naïve cells. The breakpoint was in NS5B from nt 9338 to nt 8517 (Rec#21) (Figure 3); this recombination took place in a region where 11 of 12 consecutive bases were conserved. Depending on the primers used, wild-type NS5B sequence could also be amplified from these cultures. Independent confirmation of the junction site by RT-PCR excluded cross contamination between the samples with identical breakpoint.

Since four identical recombinants were observed, we also cloned this recombinant type, J6/JFH1(Rec#21), and analyzed it in reverse genetic studies. Surprisingly, the input recombinant with the duplicated region could only be detected one day after transfection, while wild-type virus was detected thereafter. A silent mutation introduced in NS4B was amplified together with the duplicated region to exclude contamination. Thus, Rec#21 apparently resulted from one recombination event leading to a transient state, which was rapidly followed by a second recombination event leading to wild-type J6/JFH1 sequence. The presence of wild-type NS5B sequence also in the original cultures was in accordance with Rec#21 representing a transient state.

Thus, efficient recombination was demonstrated also in the 3′-end of the HCV genome. In co-transfections unbiased by the selection of HCV isolates [both J6/JFH1Δ5′ and J6/JFH1/3′X carried the complete J6/JFH1 ORF], a longer stretch of conserved nucleotides seemed to be preferred over the NS2 region for the recombination breakpoint.

## Discussion

In this study, efficient HCV RNA recombination leading to robust virus production was demonstrated in cell culture. Most recombination events were non-homologous with large in-frame insertions of up to 2400 nucleotides, while fewer homologous events were identified. Almost all recombinants identified from replication defective genomes were of different nature, and we thus found no strong site specificity. Further, recombination occurred most efficiently between isolates of the same genotype. Most identified recombinants maintained at least one complete copy of each HCV protein and many recombinants carried two copies of one or more genes. It remains to be determined whether such duplications could produce two different functional protein copies, e.g. leading to viral particles carrying envelope proteins of different isolates or give any advantage to the virus. Only one recombinant type did not have at least one intact copy of all HCV genes (Rec#21). Though this recombinant type had an internal junction in NS5B, it carried an intact globular finger-palm-thumb structure followed by duplicated sequence and finally the C-terminal membrane anchor [36].

Interestingly, HCV RNA recombination did not depend on HCV replication as co-transfection of two replication incompetent genomes led to productive recombination (Rec#6-14 and #17-21). Further, the frequencies of recombination and the time until spread of infection in culture did not seem to differ between co-transfections with and without replication competent genomes. A non-replicative mechanism for HCV recombination is in agreement with findings in cell culture for the related BVDV [23] and for poliovirus [22]. This type of recombination was shown primarily to take place at single-stranded RNA structures [37], and it is hypothesized to occur through endoribonucleolytic cleavage and subsequent ligation of 3′-phosphate and 5′-hydroxyl partners. It remains to be determined by which mechanism(s) HCV recombination occurs in patients. The replicative copy-choice mechanism has previously been favored, since it is straightforward to envision how this strategy could produce the homologous recombinants observed *in vivo*. Accordingly, a model that could explain the generation of the 2k/1b recombinant from St. Petersburg by template switching was previously suggested [38]. Here we demonstrated that homologous recombinants could be produced through a non-replicative mechanism (Rec#9 and Rec#17), which could represent an alternative or parallel pathway to replicative recombination *in vivo*.

After long-term passage in culture of the non-homologous recombinant J6/JFH1ΔE1E2(Rec#1) a more fit variant emerged, replacing the original replicating genome and leading to higher viral titers. This new recombinant resulted from a second recombination event and carried a duplication of only 186 nts compared to the original 1065 nts. Since the original recombinant was cloned and the second event occurred after a new transfection and subsequent cell-free passages, recombination must have occurred from the same genome or among genomes with identical structures and sequentially led to a more fit variant with a smaller insertion. Similar deletions of heterologous sequences have been observed in cytopathogenic BVDV genomes with heterologous sequences [39], and HCV genomes with inserted reporter genes in cell culture and *in vivo* [30], reflecting the virus ability to evolve and increase its fitness. Non-homologous recombinants have not been observed in patients [9]–[11], potentially due to strong fitness selection for homologous recombinants. However, non-homologous recombinants could represent precursors to more fit homologous recombinants through sequential recombination events, as we observed in reverse genetic experiments with J6/JFH1(Rec#21).

Co-transfections with two genomes of the same genotype led to productive recombination events in 22 of 25 experiments (86%) or 0.0016% of cells, whereas only 2 out of 18 (11%) or 0.0001% of cells in intergenotypic experiments led to productive recombination. Except for two cases of homologous recombination, all identified events were non-homologous. Reiter et al. previously described homologous recombination in the HCV replicon system [25]. However, since duplicated regions generated by non-homologous recombination between fragments of the same isolate could be obscured in direct sequencing from PCR products, non-homologous recombinants could possibly also have been occurring in that study. The recombination frequency in the replicon-based study ranged from one event per 3,000 to 30,000 cells, depending on the length of the genomic region available for recombination, or 0.003 to 0.03% of cells replicating wild-type replicons in parallel experiments [25]. This was slightly higher than frequencies observed in the present study, however in the replicon system, selection could allow less fit recombinants to survive and some recombination events might be compatible with replication but not with the complete viral life-cycle. In a study of cells infected with a non-cytopathogenic BVDV strain, which were subsequently transfected with a defective cytopathogenic genome, recombination events were observed in 33–58% of cultures when electroporated cells were plated in 24-well format, or roughly equivalent to one event per 0.001% of cells (assuming around 10^5^ cells per culture) [23]. This was in the range of what was observed in the present study on HCV. A notable difference, however, is that for BVDV this occurred for a viable genome, while observation of similar recombination frequencies for HCV depended on two non-viable genomes. A direct comparison of frequencies is complicated, since recombination is thought to be affected by the length of the genomic region available for recombination [25], replication capacity and constraints on genome organization of productive recombinants. Studies with poliovirus and BVDV previously showed that the frequency of homologous recombination decreased with decreasing sequence homology between the RNA molecules [21], [39], and that non-homologous recombination was the most frequent for recombination between different BVDV strains.

In this study, productive recombination more often took place between isolates of the same HCV genotype. The identification of several recombination events at a conserved nucleotide sequence in NS5B supports the importance of similar sequences for recombination to occur. Another explanation could be the higher functional compatibility between proteins of the same genotype expressed by the recombined RNA. The lack of sequence conservation at a number of recombination sites (Figure 3) indicates that sequence similarity is not a prerequisite for non-homologous recombination to occur. On the other hand, the high frequency of ambiguous nucleotides in recombination sites in this study (residues around the recombination site that are identical in the two parental sequences; Figure 3), indicates a role for primary sequences in dictating junction sites. Random joining would leave one ambiguous nucleotide in one of four recombination events, two ambiguous nucleotides in one of 16 events etc. Thus, the frequency of ambiguous nucleotides in cross over sites in this study is higher than expected. The low frequency of intergenotypic recombination events identified in this study is in some contradiction to the ratio of inter- and intragenotypic recombinants identified in patients [10]. However, since intragenotypic recombinants by nature are harder to define, their existence could be underrepresented in the literature.

The recombination frequency calculations from the replicon study indicated that no recombination hotspots are present in the HCV genome [25]. This is in agreement with our findings that productive HCV recombination in the infectious cell culture system is not restricted to certain regions of the genome. However, several cases of recombination between two nearly identical 12 nt stretches in NS5B indicated some preference for conserved sequences. Interestingly, the experimental setup in the replicon study did not allow recombination to occur at this potential hotspot [25]. Recombination site specificity remains to be fully investigated in the absence of constraints using identical HCV isolates covering the entire genome. It could be speculated that some restrictions on recombination sites could apply at least to non-homologous recombination. Interestingly, all recombination sites identified in this study fall in regions where recombination of natural strains was also described [10].

All recombination events identified in the present study led to joining of viral RNA fragments. While insertion of cellular sequences has been reported for several other viruses [4], [40]–[42], and is an important regulatory process for cytopathogenicity of the related BVDV [40], [43], this has not been reported for HCV in vivo. However, by cell culture transfection of deletion mutants of stem loop I of the HCV 5′UTR, we previously recovered viable genomes that acquired RNA stem loop structures derived from viral or host sequences compensating for the deletion [27]. Now knowing that replication independent recombination is possible for HCV, these variants could have arisen by such a mechanism.

Non-homologous recombination could initiate important evolutionary steps in generation of novel types of viral genomes or cause diversity in genome regions tolerating insertions and deletions. Such productive non-homologous recombination events might potentially be followed by another recombination event to get rid of duplicate fitness-lowering sequences. The importance of RNA recombination for the evolution of RNA viruses is well documented [19], and many recently emerged human diseases are caused by viruses that display active recombination or reassortment [1], [5]. The presence of reverse transcriptase could even fix such sequences in the cellular genome [44]. Thus, RNA recombination could have played an important role in cellular and viral genetic evolution.

The prevalence of HCV recombinants in patients is relatively low, which could in part be caused by the super-infection exclusion principle [45], [46], which would reduce the chance of having two different HCV strains replicating in the same cell. *In vivo*, the amount of replicating RNA is further expected to be much lower than the amounts of RNA present after co-transfection *in vitro*. Thus, the recombination frequency reported here could well be overrepresented compared to the *in vivo* setting. Further, fitness of novel recombinants *in vivo* should be high for the recombinant to eventually dominate over the parental strains. In a treatment setting this might however be accomplished, e.g. if parental genomes each carried resistance to one of two antiviral compounds in a combination therapy, with recombination leading to a double-resistant recombinant genome. Subgenomic deletion mutants [14], [15] are naturally occurring in patients and are similar in structure to the JFH1ΔE1E2 construct used in this study. These could therefore constitute a reservoir of independent genomes that could potentially recombine with the wild-type to generate treatment-resistant or otherwise high-fitness genomes. With an increased knowledge on HCV recombination, better diagnosis of clinically important recombinants could become available, thereby facilitating selection of optimal therapeutic regimens for the patients. Our findings shed new light on how HCV recombination could occur in patients. Further, viral recombination might be an important escape mechanism to specific antiviral therapy in general, which could be important to consider in design of treatment regimens for certain viruses.

## Materials and Methods

### Plasmids

The HCV plasmids pJFH1ΔE1E2 [29], pJ6/JFH1 [28], pJ6/JFH1-GND [28], pJ6CF [47], pH77C [48], pHC-TN [49], pJ4L6S [50], pS52 [26] and pED43 [26] were previously described. Introduction of single mutations and construction of pJFH1Δ5′, pJ6/JFH1Δ5′, pJ6_1–7666_, pJ6/JFH1Δ(5′-p7), pJ6/JFH1Δ(5′-NS4A), pJ6/JFH1Δ(5′-NS4B), pJ6/JFH1Δ(5′-NS5A), pJ6/JFH1/3′X, pJ6/JFH1ΔE1E2(Rec#1), pJ6/JFH1(Rec#10), pJ6/JFH1(Rec#21), pJFH1Δ5′-RlucΔ40 and pJ6/JFH1-GND-RlucΔ40 was done using standard fusion PCR and cloning methods. The J6/JFH1/3′X mutant contained a fragment of human cAMP-dependent protein kinase mRNA, replacing the HCV 3′X region. The complete HCV sequence of final plasmid preparations was confirmed.

### Culturing, transfection, infection and evaluation of cell cultures

Culturing of Huh7.5 hepatoma cells was done as previously described [51]. One day before transfection or infection, 4×10^5^ cells per well were plated in six-well plates. Before RNA transcription, plasmids were linearized with *XbaI* to generate the HCV 3′-end. To produce Δ3′ transcripts (Figure 1), linearization was done using EcoRV (nt 7764) for pJ6CF, NotI (nt 9221) for pH77C and pHC-TN, AflII (nt 9399) for pJ4L6S, NotI (nt 8549) for pS52 and KpnI (nt 9014) for pED43. Shorter truncated versions of pJ6CF were generated using ClaI (nt 709), BsiWI (nt 1345), SalI (nt 2408), BsaBI (nt 2565), NdeI (nt 2973) or AleI (nt 3480). In addition, digestion with XbaI was performed to avoid influence of minus-strand synthesis from a reverse T7 promoter. *In vitro* transcription of RNA was performed as previously described [35]. To exclude that recombination during in vitro transcription led to emergence of recombinant viral genomes, each transcript was synthesized separately. For transfections, 1.25 µg RNA of each construct (a total of 2.5 µg in co-transfections, as estimated by gel-electrophoresis) were incubated with 5 µL Lipofectamine2000 (Invitrogen) in 500 µL Opti-MEM (Invitrogen) for 20 min at room temperature. Cells were incubated with transfection complexes for 16–24 hours in growth medium or in pure Opti-MEM. For infection experiments, cells were inoculated with virus-containing supernatant for 16–24 hours.

Cell cultures were split every 2–3 days and monitored by immunostaining using mouse anti-HCV-Core-protein monoclonal antibody (B2, Anogen) or anti-NS5A 9E10 [28] as previously described [35], [51]. Supernatants collected during experiments were sterile-filtered and stored at −80°C. HCV RNA titers were determined as previously described [51]. Infectivity titers were determined by adding 100 µL of triplicate sample dilutions (diluted 1∶2 or more) to 6×10^3^ Huh7.5 cells/well plated 24 hours before infection on poly-D-lysine-coated 96-well plates (Nunc). Cells were fixed 48 hours post-infection and immunostained for HCV following a previously established protocol [51] using anti-NS5A 9E10 as primary antibody [28]. FFU were defined by clusters of infected cells separated by at least two uninfected cells. The number of FFU was determined by manual counting or by using an automated counter (ImmunoSpot Series 5 UV Analyzer, CTL Europe GmbH) with customized software, as previously described [26], [52].

To analyze recombination frequency, cells were split 18 hours after transfection and 7000 cells were plated per well in 96-well format. Starting day 5, cells were split every 2–3 days using split ratios adjusted to ensure >50% confluency in all wells throughout the experiment. At each split a replica 96-well plate was plated and incubated for 2–3 days before staining as described above for infectivity titration. In this experiment, single infected cells were counted using the ImmunoSpot Series 5 UV Analyzer.

### Luciferase translation assay

For luciferase assays, RNA was transfected into 10^5^ Huh7.5 cells/well of 24-well plates. At indicated time points, cells were lysed for 15 min according to the Renilla Luciferase Assay System (Promega) protocol, and luciferase signals were measured in 5 replicates using optical bottom 96 well plates on a FluoStarOptima (BMG) plate reader.

### Sequence determination of culture-derived HCV

HCV RNA was extracted from culture supernatant using High pure viral nucleic acid kit (Roche). For direct sequencing of the complete HCV ORF, reverse transcription, 1^st^ round PCR covering the entire ORF and 12 overlapping 2^nd^ round PCR amplifications were performed as previously described for J6/JFH1 [51]. For J4L6S and S52 intergenotypic recombination events, primers designed for the corresponding JFH1-based recombinants were used [34], [51]. Non-homologous recombination events that resulted in duplicated primer binding sites for the 2^nd^ round PCR could not be identified using the direct sequencing approach. In these cases, additional 2^nd^ round PCRs were set up with forward primers downstream of reverse primers (inverted primer sets), to specifically amplify the region containing a non-homologous recombination breakpoint with duplicated sequence. For supernatants originating from co-transfection of different HCV isolates, the initial 12 amplicon ORF direct sequencing was used to determine in which region such primers should be designed. For supernatants originating from co-transfection of RNA from the same HCV isolate, a scanning approach was used, in which inverted primer pairs placed for each ∼500 nts were tested positive or negative by PCR. Amplified PCR bands were sequenced to identify breakpoints of non-homologous recombination. In selected cases, and in cases where the recombination site could not be uniquely identified by direct sequencing, PCR products were TOPO-cloned (Invitrogen) and sequenced.

The occurrence of sequential recombination events for Rec#1 over time was monitored by PCR using primers JF1848 (CTGTGTGTGGCCCAGTGTAC) and 2763R_J6 (AGCGTGAGCCCTGACGAAGTACGG) on cDNA. The amplified product sizes varied according to the recombinant and thereby allowed differentiation. Sequence analysis was performed with Sequencher (Gene Codes Corp.).

### Control experiments

As a control for correct identification of recombinant junctions, independent RNA extraction and RT-PCR were done on supernatant of selected recombinants (Rec#1, Rec#17 and all four cultures leading to Rec#21). This confirmed the identified breakpoints. It was further confirmed that recombinant-specific PCR products could be amplified from supernatant-derived cDNA and from cloned recombinant plasmids but not from pJ6/JFH1 using inverted primer sets JF2845 (CACCCCCGGGTATAAGACC)/2111R_JFH1 (TGTACGTCCACGATGTTCTGGTG) (Rec#1) or JF1848 and the junction-specific reverse primer Rec10_R (CGTGCACAGGTGCGTCATAGGCTCCTATCTGGCCATGCACAG) (Rec#10). To exclude in vitro introduced recombination during T7-driven transcription or during reverse transcription after RNA extraction from supernatant, RNA produced by T7 transcription was subjected to 3 sequential rounds of DNAseI (Fermentas) digestion using the RNeasy kit (Qiagen), mixed to yield combinations of 5′ and 3′ partners that previously led to successful recombination in cell culture, diluted to 50 pg (equivalent to around 10^7^ copies) and subjected to RT-PCR. PCR amplification using inverted primer sets JF2845/2111R_JFH1 on J6CF and JFH1ΔE1E2 RNA (Rec#1), JF1848 and the junction-specific reverse primer Rec10_R on J6CF and JFH1ΔE1E2 RNA (Rec#10), or inverted primer sets JF8806 (CAGATACTACCTGACCAGAGAC)/JR8688 (TCCGTGAAGGCTCTCAGGTTC) on J6/JFH1/3′X and J6/JFH1(Δ5′-NS5A) RNA (Rec#21), did not lead to the specific amplicons that were observed for the cloned plasmids of the respective recombinants. Further, the viable phenotypes of all cloned recombinants and the fact that all recombination events identified led to in-frame recombinant ORFs supported that RT-PCR-induced artifacts were not misleading our conclusions.

## References

[1] Simon-Loriere E, Holmes EC (2011) Why do RNA viruses recombine? Nature reviews Microbiology 9: 617–626. 21725337 10.1038/nrmicro2614 PMC3324781

[2] Malim MH, Emerman M (2001) HIV-1 sequence variation: drift, shift, and attenuation. Cell 104: 469–472. 11239404 10.1016/s0092-8674(01)00234-3

[3] Nora T, Charpentier C, Tenaillon O, Hoede C, Clavel F, et al (2007) Contribution of recombination to the evolution of human immunodeficiency viruses expressing resistance to antiretroviral treatment. Journal of virology 81: 7620–7628. 17494080 10.1128/JVI.00083-07 PMC1933369

[4] Khatchikian D, Orlich M, Rott R (1989) Increased viral pathogenicity after insertion of a 28S ribosomal RNA sequence into the haemagglutinin gene of an influenza virus. Nature 340: 156–157. 2544809 10.1038/340156a0

[5] Hahn CS, Lustig S, Strauss EG, Strauss JH (1988) Western equine encephalitis virus is a recombinant virus. Proceedings of the National Academy of Sciences of the United States of America 85: 5997–6001. 3413072 10.1073/pnas.85.16.5997 PMC281892

[6] Kew O, Morris-Glasgow V, Landaverde M, Burns C, Shaw J, et al (2002) Outbreak of poliomyelitis in Hispaniola associated with circulating type 1 vaccine-derived poliovirus. Science 296: 356–359. 11896235 10.1126/science.1068284

[7] Becher P, Orlich M, Thiel HJ (2001) RNA recombination between persisting pestivirus and a vaccine strain: generation of cytopathogenic virus and induction of lethal disease. Journal of virology 75: 6256–6264. 11413291 10.1128/JVI.75.14.6256-6264.2001 PMC114347

[8] Simmonds P (2006) Recombination and selection in the evolution of picornaviruses and other Mammalian positive-stranded RNA viruses. Journal of virology 80: 11124–11140. 16956935 10.1128/JVI.01076-06 PMC1642140

[9] Gottwein JM, Bukh J (2008) Cutting the gordian knot-development and biological relevance of hepatitis C virus cell culture systems. Advances in virus research 71: 51–133. 18585527 10.1016/S0065-3527(08)00002-X

[10] Morel V, Fournier C, Francois C, Brochot E, Helle F, et al (2011) Genetic recombination of the hepatitis C virus: clinical implications. Journal of viral hepatitis 18: 77–83. 21235686 10.1111/j.1365-2893.2010.01367.x

[11] Gonzalez-Candelas F, Lopez-Labrador FX, Bracho MA (2011) Recombination in hepatitis C virus. Viruses 3: 2006–2024. 22069526 10.3390/v3102006 PMC3205392

[12] Simmonds P, Bukh J, Combet C, Deleage G, Enomoto N, et al (2005) Consensus proposals for a unified system of nomenclature of hepatitis C virus genotypes. Hepatology 42: 962–973. 16149085 10.1002/hep.20819

[13] Kalinina O, Norder H, Mukomolov S, Magnius LO (2002) A natural intergenotypic recombinant of hepatitis C virus identified in St. Petersburg. Journal of virology 76: 4034–4043. 11907242 10.1128/JVI.76.8.4034-4043.2002 PMC136067

[14] Pacini L, Graziani R, Bartholomew L, De Francesco R, Paonessa G (2009) Naturally occurring hepatitis C virus subgenomic deletion mutants replicate efficiently in Huh-7 cells and are trans-packaged in vitro to generate infectious defective particles. Journal of virology 83: 9079–9093. 19587042 10.1128/JVI.00308-09 PMC2738267

[15] Yagi S, Mori K, Tanaka E, Matsumoto A, Sunaga F, et al (2005) Identification of novel HCV subgenome replicating persistently in chronic active hepatitis C patients. Journal of medical virology 77: 399–413. 16173026 10.1002/jmv.20469

[16] Palmer BA, Moreau I, Levis J, Harty C, Crosbie O, et al (2012) Insertion and recombination events at hypervariable region 1 over 9.6 years of hepatitis C virus chronic infection. The Journal of general virology 93: 2614–2624. 22971825 10.1099/vir.0.045344-0

[17] Manns MP, Wedemeyer H, Cornberg M (2006) Treating viral hepatitis C: efficacy, side effects, and complications. Gut 55: 1350–1359. 16905701 10.1136/gut.2005.076646 PMC1860034

[18] Sarrazin C, Hezode C, Zeuzem S, Pawlotsky JM (2012) Antiviral strategies in hepatitis C virus infection. Journal of hepatology 56 Suppl 1: S88–100. 22300469 10.1016/S0168-8278(12)60010-5

[19] Lai MM (1992) RNA recombination in animal and plant viruses. Microbiological reviews 56: 61–79. 1579113 10.1128/mr.56.1.61-79.1992 PMC372854

[20] Worobey M, Holmes EC (1999) Evolutionary aspects of recombination in RNA viruses. The Journal of general virology 80(Pt 10): 2535–2543. 10573145 10.1099/0022-1317-80-10-2535

[21] Kirkegaard K, Baltimore D (1986) The mechanism of RNA recombination in poliovirus. Cell 47: 433–443. 3021340 10.1016/0092-8674(86)90600-8 PMC7133339

[22] Gmyl AP, Belousov EV, Maslova SV, Khitrina EV, Chetverin AB, et al (1999) Nonreplicative RNA recombination in poliovirus. Journal of virology 73: 8958–8965. 10516001 10.1128/jvi.73.11.8958-8965.1999 PMC112927

[23] Gallei A, Pankraz A, Thiel HJ, Becher P (2004) RNA recombination in vivo in the absence of viral replication. Journal of virology 78: 6271–6281. 15163720 10.1128/JVI.78.12.6271-6281.2004 PMC416528

[24] Gao F, Nainan OV, Khudyakov Y, Li J, Hong Y, et al (2007) Recombinant hepatitis C virus in experimentally infected chimpanzees. The Journal of general virology 88: 143–147. 17170446 10.1099/vir.0.82263-0

[25] Reiter J, Perez-Vilaro G, Scheller N, Mina LB, Diez J, et al (2011) Hepatitis C virus RNA recombination in cell culture. Journal of hepatology 55: 777–783. 21334392 10.1016/j.jhep.2010.12.038

[26] Gottwein JM, Scheel TK, Callendret B, Li YP, Eccleston HB, et al (2010) Novel infectious cDNA clones of hepatitis C virus genotype 3a (strain S52) and 4a (strain ED43): genetic analyses and in vivo pathogenesis studies. Journal of virology 84: 5277–5293. 20200247 10.1128/JVI.02667-09 PMC2863810

[27] Li YP, Gottwein JM, Scheel TK, Jensen TB, Bukh J (2011) MicroRNA-122 antagonism against hepatitis C virus genotypes 1–6 and reduced efficacy by host RNA insertion or mutations in the HCV 5′ UTR. Proceedings of the National Academy of Sciences of the United States of America 108: 4991–4996. 21383155 10.1073/pnas.1016606108 PMC3064388

[28] Lindenbach BD, Evans MJ, Syder AJ, Wolk B, Tellinghuisen TL, et al (2005) Complete replication of hepatitis C virus in cell culture. Science 309: 623–626. 15947137 10.1126/science.1114016

[29] Wakita T, Pietschmann T, Kato T, Date T, Miyamoto M, et al (2005) Production of infectious hepatitis C virus in tissue culture from a cloned viral genome. Nature medicine 11: 791–796. 10.1038/nm1268 PMC2918402 15951748

[30] Gottwein JM, Jensen TB, Mathiesen CK, Meuleman P, Serre SB, et al (2011) Development and Application of Hepatitis C Reporter Viruses with Genotype 1 to 7 Core-Nonstructural Protein 2 (NS2) Expressing Fluorescent Proteins or Luciferase in Modified JFH1 NS5A. Journal of virology 85: 8913–8928. 21697486 10.1128/JVI.00049-11 PMC3165809

[31] Murayama A, Weng L, Date T, Akazawa D, Tian X, et al (2010) RNA polymerase activity and specific RNA structure are required for efficient HCV replication in cultured cells. PLoS pathogens 6: e1000885. 20442786 10.1371/journal.ppat.1000885 PMC2861710

[32] Gottwein JM, Scheel TK, Jensen TB, Lademann JB, Prentoe JC, et al (2009) Development and characterization of hepatitis C virus genotype 1–7 cell culture systems: role of CD81 and scavenger receptor class B type I and effect of antiviral drugs. Hepatology 49: 364–377. 19148942 10.1002/hep.22673

[33] Pietschmann T, Kaul A, Koutsoudakis G, Shavinskaya A, Kallis S, et al (2006) Construction and characterization of infectious intragenotypic and intergenotypic hepatitis C virus chimeras. Proceedings of the National Academy of Sciences of the United States of America 103: 7408–7413. 16651538 10.1073/pnas.0504877103 PMC1455439

[34] Scheel TK, Gottwein JM, Carlsen TH, Li YP, Jensen TB, et al (2011) Efficient culture adaptation of hepatitis C virus recombinants with genotype-specific core-NS2 by using previously identified mutations. Journal of virology 85: 2891–2906. 21177811 10.1128/JVI.01605-10 PMC3067958

[35] Scheel TK, Gottwein JM, Jensen TB, Prentoe JC, Hoegh AM, et al (2008) Development of JFH1-based cell culture systems for hepatitis C virus genotype 4a and evidence for cross-genotype neutralization. Proceedings of the National Academy of Sciences of the United States of America 105: 997–1002. 18195353 10.1073/pnas.0711044105 PMC2242719

[36] Lesburg CA, Cable MB, Ferrari E, Hong Z, Mannarino AF, et al (1999) Crystal structure of the RNA-dependent RNA polymerase from hepatitis C virus reveals a fully encircled active site. Nature structural biology 6: 937–943. 10504728 10.1038/13305

[37] Austermann-Busch S, Becher P (2012) RNA Structural Elements Determine Frequency and Sites of Nonhomologous Recombination in an Animal Plus-Strand RNA Virus. Journal of virology 86: 7393–402. 22532677 10.1128/JVI.00864-12 PMC3416315

[38] Kalinina O, Norder H, Magnius LO (2004) Full-length open reading frame of a recombinant hepatitis C virus strain from St Petersburg: proposed mechanism for its formation. The Journal of general virology 85: 1853–1857. 15218169 10.1099/vir.0.79984-0

[39] Gallei A, Orlich M, Thiel HJ, Becher P (2005) Noncytopathogenic pestivirus strains generated by nonhomologous RNA recombination: alterations in the NS4A/NS4B coding region. Journal of virology 79: 14261–14270. 16254361 10.1128/JVI.79.22.14261-14270.2005 PMC1280241

[40] Meyers G, Rumenapf T, Thiel HJ (1989) Ubiquitin in a togavirus. Nature 341: 491. 10.1038/341491a0 2552321

[41] Monroe SS, Schlesinger S (1983) RNAs from two independently isolated defective interfering particles of Sindbis virus contain a cellular tRNA sequence at their 5′ ends. Proceedings of the National Academy of Sciences of the United States of America 80: 3279–3283. 6304704 10.1073/pnas.80.11.3279 PMC394024

[42] Munishkin AV, Voronin LA, Chetverin AB (1988) An in vivo recombinant RNA capable of autocatalytic synthesis by Q beta replicase. Nature 333: 473–475. 2453805 10.1038/333473a0

[43] Becher P, Tautz N (2011) RNA recombination in pestiviruses: cellular RNA sequences in viral genomes highlight the role of host factors for viral persistence and lethal disease. RNA biology 8: 216–224. 21358277 10.4161/rna.8.2.14514

[44] Geuking MB, Weber J, Dewannieux M, Gorelik E, Heidmann T, et al (2009) Recombination of retrotransposon and exogenous RNA virus results in nonretroviral cDNA integration. Science 323: 393–396. 19150848 10.1126/science.1167375

[45] Schaller T, Appel N, Koutsoudakis G, Kallis S, Lohmann V, et al (2007) Analysis of hepatitis C virus superinfection exclusion by using novel fluorochrome gene-tagged viral genomes. Journal of virology 81: 4591–4603. 17301154 10.1128/JVI.02144-06 PMC1900174

[46] Tscherne DM, Evans MJ, von Hahn T, Jones CT, Stamataki Z, et al (2007) Superinfection exclusion in cells infected with hepatitis C virus. Journal of virology 81: 3693–3703. 17287280 10.1128/JVI.01748-06 PMC1866098

[47] Yanagi M, Purcell RH, Emerson SU, Bukh J (1999) Hepatitis C virus: an infectious molecular clone of a second major genotype (2a) and lack of viability of intertypic 1a and 2a chimeras. Virology 262: 250–263. 10489358 10.1006/viro.1999.9889

[48] Yanagi M, Purcell RH, Emerson SU, Bukh J (1997) Transcripts from a single full-length cDNA clone of hepatitis C virus are infectious when directly transfected into the liver of a chimpanzee. Proceedings of the National Academy of Sciences of the United States of America 94: 8738–8743. 9238047 10.1073/pnas.94.16.8738 PMC23104

[49] Sakai A, Takikawa S, Thimme R, Meunier JC, Spangenberg HC, et al (2007) In vivo study of the HC-TN strain of hepatitis C virus recovered from a patient with fulminant hepatitis: RNA transcripts of a molecular clone (pHC-TN) are infectious in chimpanzees but not in Huh7.5 cells. Journal of virology 81: 7208–7219. 17409145 10.1128/JVI.01774-06 PMC1933310

[50] Yanagi M, St Claire M, Shapiro M, Emerson SU, Purcell RH, et al (1998) Transcripts of a chimeric cDNA clone of hepatitis C virus genotype 1b are infectious in vivo. Virology 244: 161–172. 9581788 10.1006/viro.1998.9092

[51] Gottwein JM, Scheel TK, Hoegh AM, Lademann JB, Eugen-Olsen J, et al (2007) Robust hepatitis C genotype 3a cell culture releasing adapted intergenotypic 3a/2a (S52/JFH1) viruses. Gastroenterology 133: 1614–1626. 17983807 10.1053/j.gastro.2007.08.005

[52] Scheel TK, Gottwein JM, Mikkelsen LS, Jensen TB, Bukh J (2011) Recombinant HCV variants with NS5A from genotypes 1–7 have different sensitivities to an NS5A inhibitor but not interferon-alpha. Gastroenterology 140: 1032–1042. 21111742 10.1053/j.gastro.2010.11.036

